# Attachment Anxiety and Disordered Eating Among Chinese Young Women: The Mediation of Different Negative Emotions

**DOI:** 10.3390/bs16091515

**Published:** 2026-08-28

**Authors:** Chenran Li, Xiangyi Liang, Shufan Xu, Panpan Zheng, Zhenyong Lyu

**Affiliations:** School of Education, Yangzhou University, Yangzhou 225000, China; chenranli12@163.com (C.L.); xiangyiliang12@163.com (X.L.); shufanxu07@126.com (S.X.); 007530@yzu.edu.cn (P.Z.)

**Keywords:** attachment anxiety, disordered eating, negative emotions, young women

## Abstract

Disordered eating is increasingly prevalent among young women, yet the psychological mechanisms underlying these behaviors remain insufficiently understood. This cross-sectional study examined the associations between attachment anxiety and three distinct patterns of disordered eating: uncontrolled eating (UE), emotional eating (EE), and cognitive restraint (CR). Additionally, we investigated the mediating roles of three negative emotions (i.e., depression, anxiety, and stress) in these relationships. Data were collected from 578 young Chinese women (Mean age = 19.48 years) using self-report scales assessing attachment anxiety, negative emotions, and disordered eating behaviors. Path analysis revealed that attachment anxiety had significant direct positive predictive effects on UE and EE but did not significantly predict CR. Anxiety and stress significantly mediated the relationship between attachment anxiety and both UE and EE, whereas depression did not exhibit a significant mediating effect. Furthermore, the mediating effects of the three negative emotions on the association between attachment anxiety and CR did not reach statistical significance, indicating that the observed patterns are not uniform across different disordered eating behaviors. These findings highlight the differential roles of negative emotions in the association between attachment anxiety and disordered eating behaviors among young women.

## 1. Introduction

Disordered eating refers to various types of abnormal eating-related behaviors that often fail to meet the clinical diagnostic criteria for an eating disorder, while still affecting physiological and psychological well-being (Gonçalves & Cesar Machado, 2024). Based on the Three-Factor Eating Questionnaire, such behaviors are classified into three categories: emotional eating (EE), uncontrolled eating (UE), and cognitive restraint (CR) (Karlsson et al., 2000). EE refers to excessive food intake triggered by emotions (Van Strien, 2018); UE refers to overeating with a subjective sense of loss of control; and CR refers to deliberate food restriction to regulate body shape and weight (Bryant et al., 2019). A detailed description of this questionnaire is provided in Section 2. Young women, particularly female university students, represent a high-risk group for disordered eating (Alhaj et al., 2022; Yu et al., 2018). In China, the overall prevalence of disordered eating among women is 7.04%, increasing to 11.24% among young women (Yao et al., 2021). Disordered eating is linked to significant mental health consequences, including psychological distress (Kärkkäinen et al., 2018), comorbidity of depression and anxiety (Tan et al., 2023), and suicide-related mortality (Semchishen et al., 2026). Therefore, identifying psychological risk factors and underlying mechanisms of disordered eating is crucial. Moreover, age and body mass index (BMI) have been shown to influence disordered eating (Hong et al., 2015) and should therefore be controlled for in analyses.

Insecure attachment has been identified as an important psychological risk factor for disordered eating (Donato et al., 2024; Rossi et al., 2022). Among the dimensions of insecure attachment, attachment anxiety may be particularly relevant to disordered eating. Attachment anxiety is characterized by a strong need for intimacy and excessive worry about abandonment or rejection in relationships (Brennan et al., 1998). Unlike attachment avoidance, which involves the denial of distress, attachment anxiety is characterized by heightened emotional reactivity, a state that more readily translates into affect-regulation behaviors such as disordered eating (Mikulincer & Shaver, 2007; Faber et al., 2018). Therefore, this study focuses specifically on attachment anxiety. Previous studies have demonstrated that attachment anxiety is closely linked to various disordered eating behaviors. A recent review revealed that attachment anxiety serves as the most stable and powerful positive predictor of EE (Nader et al., 2025). Moreover, attachment anxiety has been found to indirectly influence restrained eating through emotional reactivity (Han & Kahn, 2017), and Alexander (2017) reported a significant association between attachment anxiety and binge eating tendencies. However, the psychological mechanisms underlying how attachment anxiety translates into disordered eating remain insufficiently understood.

Attachment theory provides a framework for understanding this mechanism. Individuals with high attachment anxiety develop negative self and positive other working models, which heighten sensitivity to rejection cues and increase the tendency to interpret ambiguous interpersonal signals as threatening (Bartholomew & Horowitz, 1991; Bowlby, 1969). Such hyperactivation of the attachment system may contribute to persistent worries and heightened emotional reactivity, thereby increasing negative affect (Mikulincer & Shaver, 2007). From the perspective of emotion regulation theory, individuals with high attachment anxiety often experience greater emotion regulation difficulties and tend to adopt maladaptive strategies to regulate negative emotions (Brandão et al., 2023; Gross, 1998). Unlike adaptive strategies such as cognitive reappraisal, maladaptive emotion regulation strategies typically provide short-term emotional relief through immediately rewarding behaviors but fail to effectively alleviate emotional distress (Gross, 2015; Weiss et al., 2022). Disordered eating may represent a typical form of maladaptive emotion regulation, whereby individuals attempt to alleviate negative emotions through food consumption, increasing their vulnerability to disordered eating (Brytek-Matera, 2021; Prefit et al., 2019). Moreover, individuals with high attachment anxiety may be more likely to misinterpret negative emotions as hunger sensations, which may contribute to their vulnerability to disordered eating (Gonçalves et al., 2021; Nader et al., 2025). Therefore, negative affect may mediate the association between attachment anxiety and disordered eating.

Importantly, negative emotions are not a homogeneous construct. Lovibond and Lovibond (1995) conceptualized negative emotions as three dimensions: depression, anxiety, and stress. Within this framework, depression reflects diminished positive affect, anxiety reflects hyperarousal, and stress reflects symptoms such as irritability, nervous tension, difficulty relaxing, and agitation. Research has shown that attachment anxiety is associated with all three dimensions. Previous studies found that attachment anxiety predicts depressive and anxiety symptoms (Blake et al., 2025; Vowels et al., 2022). A meta-analysis further confirmed a significant association between attachment anxiety and depressive symptoms (Zheng et al., 2020). Read et al. (2018) pointed out that social anxiety is positively correlated with attachment anxiety, and individuals with higher attachment anxiety experience greater anxiety in social contexts due to self-doubt and fear of rejection. Kural and Özyurt (2023) found that individuals with high attachment anxiety report higher perceived stress, which may be related to hyperactivating coping strategies and negative attributional styles (Simpson & Rholes, 2017). However, depression, anxiety, and stress reflect distinct psychological processes. Examining negative emotions as a global construct may obscure the unique contributions of each dimension to disordered eating.

Furthermore, different negative emotions may show differential associations with specific types of disordered eating. For example, higher perceived stress has been linked to bulimic tendencies and uncontrolled eating (UE) (Sporea et al., 2025), and binge eating appears primarily driven by elevated stress rather than depression or anxiety alone (Cecchetto et al., 2021). Thus, stress appears to be more strongly associated with UE than depression or anxiety. Similarly, Silva et al. (2025) found that depression, anxiety, and stress were each independently associated with higher emotional eating (EE) scores, with stress showing the strongest association. Consistently, Brisotto et al. (2022) demonstrated that stress, depression, and anxiety explained 12%, 9.2%, and 4.7% of the variance in EE, respectively. Thus, stress appears to be most strongly associated with EE, though depression and anxiety also show consistent links. In contrast, cognitive restraint (CR) may involve different mechanisms. A review found that CR was positively associated with body dissatisfaction, though its association with depression was mixed (Yavorivski et al., 2026). Moreover, Lim et al. (2021) showed that depression, anxiety, and stress did not independently predict CR longitudinally, although the interaction between anxiety and stress did. Therefore, among the three negative emotions, none appear to be a robust correlate of CR. These findings suggest that different negative emotions may influence different forms of disordered eating through distinct pathways.

Overall, current research on the relationship between attachment anxiety and disordered eating remains limited. Previous studies have typically examined disordered eating as a global construct (Jin et al., 2024; Klein et al., 2022), with limited attention to whether different negative emotions contribute to different eating behaviors through distinct pathways. Moreover, prior mediation studies have primarily focused on emotion regulation (Gómez-Odriozola et al., 2026; Taube-Schiff et al., 2015), body dissatisfaction (Nasrallah et al., 2025; Valledor et al., 2024), and perfectionism (Dakanalis et al., 2014), yet most have treated emotion as a broad, overarching construct without systematically distinguishing or testing the specific mediating effects of different negative emotions.

Therefore, the present study constructed a parallel mediation model to investigate the relationship between attachment anxiety and three dimensions of disordered eating among Chinese young women, while examining the parallel mediating roles of depression, anxiety, and stress. This study may contribute to a deeper understanding of the psychological mechanisms underlying the association between attachment anxiety and disordered eating, while also clarifying the differential roles of specific negative emotions in distinct eating behaviors. These findings may inform future interventions targeting emotion-specific mechanisms. Based on existing theoretical perspectives and empirical evidence, the following hypotheses were proposed:

**Hypothesis** **1.** *Attachment anxiety positively predicts disordered eating.*

**Hypothesis** **2.** *Attachment anxiety positively predicts depression, anxiety, and stress.*

**Hypothesis** **3.** *Negative emotions mediate the relationship between attachment anxiety and disordered eating, with different negative emotions showing differential mediating effects across distinct forms of disordered eating.*

## 2. Methods

### 2.1. Participants and Procedure

An a priori power analysis was conducted using G*Power 3.1 (Heinrich-Heine-Universität Düsseldorf, Düsseldorf, Germany; Faul et al., 2009) before participant recruitment to determine the minimum sample size required for testing the hypothesized multiple mediation model. For a linear multiple regression with six predictors (f^2^ = 0.15, α = 0.05, power = 0.80), the required sample size was 98.

Participants were female undergraduate students recruited from a university in China in June 2024. A convenience sampling method was adopted, and recruitment was conducted offline at the classroom level. Specifically, the researchers contacted instructors of different undergraduate classes and obtained permission to distribute paper-based questionnaires during regular teaching periods. Before questionnaire administration, potential participants were briefly informed about the study objectives, procedures, and ethical considerations. After being fully informed of the study content and their rights as participants, students who voluntarily agreed to participate provided written informed consent and completed an anonymous questionnaire. No personally identifiable information was collected. Participants were informed that participation was entirely voluntary and that they could withdraw from the study at any stage without any adverse consequences. Upon completion of the questionnaire, participants received 5 RMB as compensation. The study was conducted in accordance with the principles of the Declaration of Helsinki and was approved by the Ethics Committee of the School of Education, Yangzhou University (Approval Number: JKY-20240307002).

A total of 607 questionnaires were distributed and collected. According to the predefined exclusion criteria (i.e., extensive missing responses or obvious invalid response patterns), 29 invalid questionnaires were excluded. The final sample consisted of 578 valid participants, yielding a valid response rate of 95.2%. Given the low overall rate of missing data, cases with excessive missing responses were handled using casewise deletion. Participants were aged between 18 and 25 years (mean = 19.48, standard deviation = 1.48). Detailed demographic information of participants is presented in Table 1.

### 2.2. Inclusion Criteria

Participants were eligible for inclusion if they met the following criteria: (1) currently enrolled undergraduate students at the university; (2) aged 18 years or older; (3) able to understand the questionnaire content and complete the questionnaire independently; and (4) willing to participate voluntarily and provide valid written informed consent.

As this study did not involve clinical diagnostic assessments, information regarding diagnoses of depression, anxiety disorders, eating disorders, or chronic illnesses was not collected, and participants were not excluded based on these conditions.

### 2.3. Measures

#### 2.3.1. Attachment Anxiety

The anxiety subscale of the Revised Adult Attachment Scale (RAAS) was used to assess individuals’ anxiety regarding abandonment and being unloved (Collins, 1996). The subscale consists of 6 items rated on a 5-point Likert scale ranging from 1 (“not at all characteristic of me”) to 5 (“very characteristic of me”). The total score ranges from 6 to 30, with higher scores indicating higher levels of attachment anxiety. The scale has been previously validated in Chinese samples (Wu et al., 2004). In the present sample, Cronbach’s α coefficient for the anxiety subscale was 0.81, indicating good internal consistency.

#### 2.3.2. Negative Emotions

The Depression, Anxiety and Stress Scale-21 (DASS-21) was used to assess three core negative emotional states: depression, anxiety, and stress (Lovibond & Lovibond, 1995). The scale consists of 21 items rated on a 4-point Likert scale ranging from 0 (“did not apply to me at all”) to 3 (“applied to me very much, or most of the time”). Each subscale contains 7 items, with scores ranging from 0 to 21. Higher scores indicate higher levels of depression, anxiety, and stress symptoms. In the present study, raw scores were used for statistical analyses, and no doubling transformation was applied. The DASS-21 has shown robust psychometric properties, including reliability, stability, and validity, within Chinese populations (Gong et al., 2010; Li et al., 2012; Luo et al., 2025). In the present sample, Cronbach’s α coefficient was 0.86 for the depression subscale, 0.82 for the anxiety subscale, 0.83 for the stress subscale, and 0.93 for the total scale, indicating good internal consistency.

#### 2.3.3. Disordered Eating Behavior

The Three-Factor Eating Questionnaire-18 (TFEQ-18) was used to assess three eating behaviors: cognitive restraint, uncontrolled eating, and emotional eating (Karlsson et al., 2000). The questionnaire consists of 18 items. The first 17 items are rated on a 4-point Likert scale ranging from 1 (“definitely false”) to 4 (“definitely true”), whereas item 18 is rated on an 8-point scale ranging from 1 (“no restraint in eating”) to 8 (“total restraint”). Following previous studies (de Lauzon et al., 2004; De Lauzon-Guillain et al., 2017; Ruggiero et al., 2026), item 18 was converted from an 8-point scale to a 4-point scale to ensure consistency across items. Raw subscale scores were then transformed to a 0–100 scale using the following formula: [(raw score − lowest possible raw score)/possible raw score range] × 100. Higher scores indicate greater levels of cognitive restraint, uncontrolled eating, and emotional eating. In this study, the Chinese version of the TFEQ-18 was administered, as established by prior validation studies (Lyu et al., 2025; Shi et al., 2011). In the present sample, Cronbach’s α coefficient was 0.76 for the cognitive restraint subscale, 0.87 for the uncontrolled eating subscale, 0.79 for the emotional eating subscale, and 0.87 for the total scale.

#### 2.3.4. Anthropometric Measurements

Participants reported their height and weight in an open-ended format on the questionnaire. Body mass index (BMI) was calculated using self-reported height and weight [BMI = weight (kg)/height (m)^2^].

### 2.4. Statistical Analysis

First, descriptive statistics and normality assessments were conducted for all measured variables using SPSS 25.0 (IBM Corp., Armonk, NY, USA). Next, Pearson correlation analyses were performed to examine the bivariate correlations among variables. Then, the Variance Inflation Factor (VIF) was calculated to assess multicollinearity. Finally, path analyses were conducted using maximum likelihood estimation in Mplus version 8.3 (Muthén & Muthén, Los Angeles, CA, USA) to test the hypothesized mediation models. Specifically, attachment anxiety was specified as the independent variable; depression, anxiety, and stress were specified as parallel mediators; and uncontrolled eating, emotional eating, and cognitive restraint were specified as dependent variables. A single-group path model was estimated, with age and BMI included as covariates. The residual covariances among the three mediators were freely estimated, as were the residual covariances among the three dependent variables. Bootstrap procedures with 5000 resamples were used to calculate the 95% bias-corrected confidence intervals (CIs) for indirect effects. An indirect effect was considered statistically significant if its 95% confidence interval did not contain zero. Standardized estimates (Est), standard errors (SE), and the lower and upper limits of the 95% confidence intervals (LLCI and ULCI, respectively) were reported for each pathway.

## 3. Results

### 3.1. Preliminary Analyses

Table 2 presents the descriptive statistics of all variables, including means, standard deviations, skewness, and kurtosis. The absolute values of skewness were below 3, and the absolute values of kurtosis were below 10 for all variables, indicating no severe violations of normality (Kline, 2020).

Table 3 presents the Pearson correlation analyses among variables. Attachment anxiety was significantly positively correlated with depression, anxiety, and stress. Moreover, attachment anxiety was also significantly positively correlated with UE, EE, and CR. Depression, anxiety, and stress were all positively associated with UE and EE. Additionally, both anxiety and stress were positively correlated with CR.

Collinearity diagnostics showed that the VIF values for age, BMI, attachment anxiety, depression, anxiety, and stress were 1.01, 1.04, 1.26, 2.87, 3.80 and 3.93, respectively. All VIF values were below the commonly accepted threshold of 5 (Hair et al., 2017), indicating no evidence of serious multicollinearity among the study variables.

### 3.2. Path Analyses

Given that this model encompassed all potential associations, it was specified as a saturated model with zero degrees of freedom. The results revealed that the model explained 15.8% of the variance in depression, 20.0% in anxiety, 20.2% in stress, 19.3% in UE, 20.9% in EE, and 6.0% in CR.

Specifically, attachment anxiety was found to positively predict depression, anxiety, stress, UE, and EE, but not CR. Furthermore, UE and EE were positively predicted by anxiety and stress. However, depression did not significantly predict any of the three disordered eating behaviors, nor did anxiety or stress predict CR.

**Figure 1:**
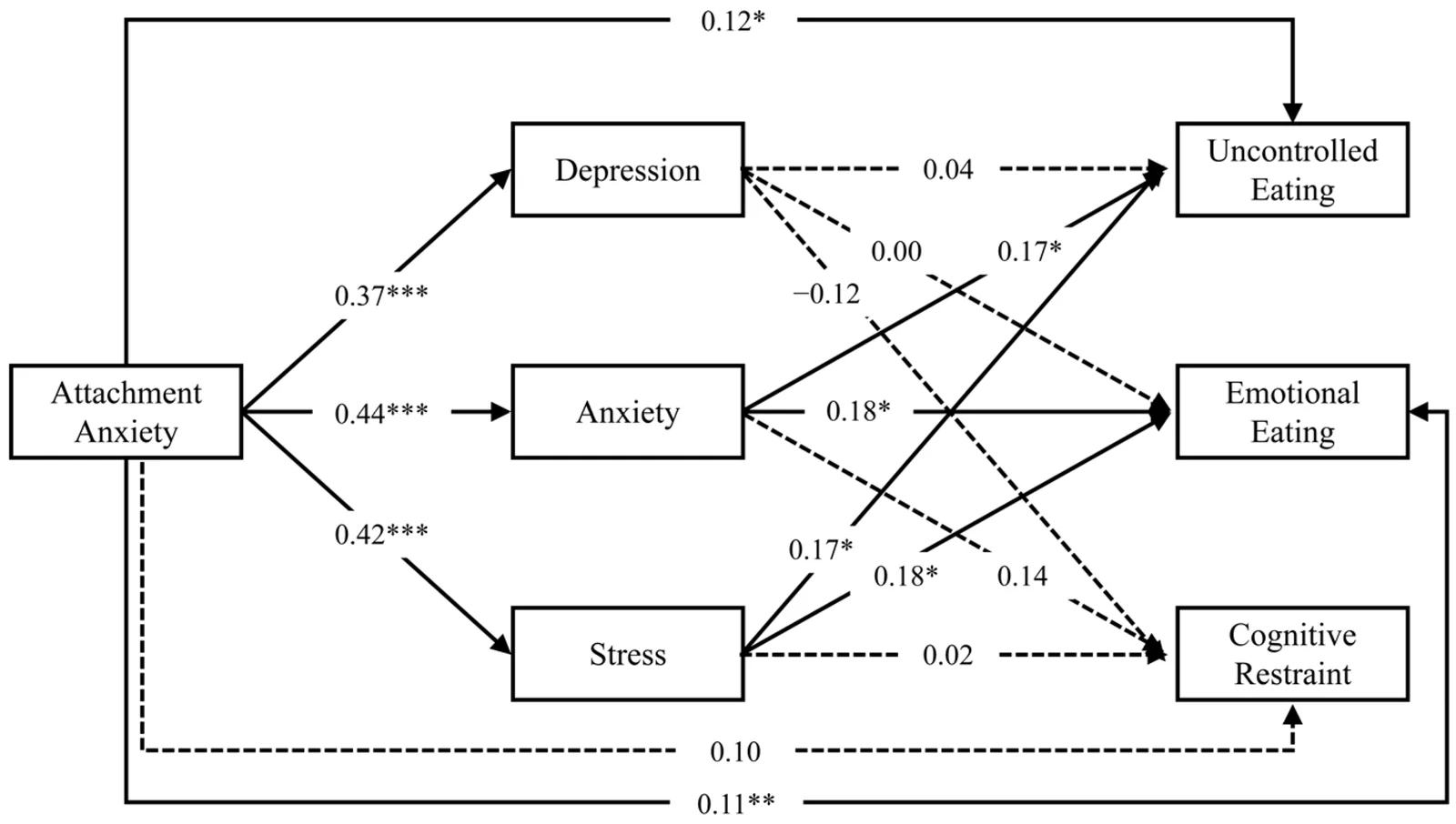
Parallel mediation model of depression, anxiety, and stress on the relationship between attachment anxiety and disordered eating in Chinese young women. Note. * *p* < 0.05, ** *p* < 0.01, *** *p* < 0.001. The solid lines represent the significant paths for *p* < 0.05. The dashed lines represent the non-significant paths.

Parallel mediation analyses were conducted to examine the mediating roles of depression, anxiety, and stress in the relationship between attachment anxiety and disordered eating: UE, EE, and CR. The analyses revealed that the relationships between attachment anxiety and both UE and EE were mediated by anxiety and stress. However, depression did not mediate the relationship between attachment anxiety and any of the disordered eating behaviors. Additionally, none of the three negative emotions significantly mediated the association between attachment anxiety and CR (see Figure 1 and Table 4).

## 4. Discussion

This study investigated the relationship between attachment anxiety and three types of disordered eating behaviors among young Chinese women and examined the parallel mediating roles of three negative emotions. The results showed that attachment anxiety had a significant positive direct predictive effect on uncontrolled eating (UE) and emotional eating (EE), which partially supported Hypothesis 1. However, attachment anxiety did not have a significant positive direct predictive effect on cognitive restraint (CR). CR is a relatively stable disposition, impervious to transient fluctuations in anxiety (Hussenoeder et al., 2021). This stability is supported by cross-sectional studies on non-clinical samples, which have consistently failed to find a significant correlation between anxiety and cognitive restraint (Aoun et al., 2019; Fekete et al., 2021; Hussenoeder et al., 2021). The different predictive effects of attachment anxiety on the three eating behaviors can be explained from the perspective of emotion regulation; individuals with high attachment anxiety typically rely on hyperactivating strategies, resulting in heightened emotional reactivity and emotion dysregulation (Mikulincer & Shaver, 2003). At the same time, because of their underdeveloped emotion regulation capacities and lack of internal self-soothing abilities, they are more inclined to rely on external modes of emotion regulation, such as eating behaviors (Maunder et al., 2017; Maunder & Hunter, 2001), manifesting as UE and EE, rather than CR, which restricts food intake. In addition, individuals with attachment anxiety are prone to misinterpreting emotional arousal as physiological hunger (Alexander & Siegel, 2013; Stapleton & Mackay, 2014), thereby triggering eating behaviors rather than CR.

Furthermore, in line with Hypothesis 2, the study found that attachment anxiety significantly and positively predicted depression, anxiety, and stress, consistent with prior study (Tammilehto et al., 2023). Attachment theory posits that individuals with high attachment anxiety often operate with an “internal working model” characterized by a “negative self-positive other” schema. They crave the security provided by close relationships yet simultaneously harbor a persistent fear of rejection and abandonment (Mikulincer & Shaver, 2007). Such individuals may view negative emotional experiences as consistent with their goal of seeking proximity and their hyperactivated attachment system, potentially leading them to focus on and even amplify these negative emotional experiences (Mikulincer & Shaver, 2019).

Hypothesis 3 proposed that negative emotions mediate the relationship between attachment anxiety and disordered eating, with different negative emotions showing differential mediating effects across the three eating behavior dimensions. The study results partially supported this hypothesis: anxiety and stress significantly mediated the relationships between attachment anxiety and both UE and EE, whereas the mediating effect of depression was not significant. Notably, none of the three negative emotions significantly mediated the relationship between attachment anxiety and CR.

Studies have shown that individuals with high attachment anxiety are more prone to experiencing negative emotions (Tammilehto et al., 2023). A core feature of attachment anxiety is persistent vigilance toward interpersonal rejection or abandonment, an urgent need for security, and the consequent state of high arousal—namely hypersensitivity to external threats and a strong demand for emotion regulation (Mikulincer & Shaver, 2007). This motivation is essentially urgent comfort-seeking (Clark et al., 2020). Anxiety and stress precisely exhibit high-arousal characteristics matching this motivation: anxiety, as an anticipatory response to future threats, and stress, as a perceived response to current demands, are both accompanied by intense physiological arousal (e.g., accelerated heart rate, elevated cortisol secretion) and psychological distress (Barlow, 2002; James et al., 2023). Such high-arousal negative emotions can directly drive individuals to adopt behaviors that rapidly alleviate negative affect, such as consuming high-sugar and high-fat foods to quickly activate the brain’s reward system (Thanarajah et al., 2019), thereby achieving rapid emotional soothing. In addition, they deplete self-control resources and weaken individuals’ ability to resist food temptations, manifesting as UE (Loth et al., 2016).

In contrast, the core features of depression are the opposite, characterized by a state of low arousal, including anhedonia and loss of interest (Horne et al., 2021). This low-arousal emotional state may not bear the high-arousal, urgent need for comfort that is unique to attachment anxiety but instead manifests more as a pattern of emotional exhaustion (Meier & Kim, 2022; Wen et al., 2025) and behavioral withdrawal (Jesulola et al., 2015). Therefore, depressive mood may not effectively transmit the motivation of attachment anxiety to eating behaviors. Meanwhile, depression is typically accompanied by diminished motivation (Grahek et al., 2019), which weakens the behavioral tendency of individuals with high attachment anxiety to seek urgent comfort through eating behaviors.

CR represents a conscious, goal-directed behavior centered on an individual’s deliberate effort to restrict food intake to control body weight or shape (Herman & Mack, 1975). Unlike UE and EE, CR involves substantial cognitive control and planning (Brytek-Matera, 2021). However, high levels of attachment anxiety (Stanton & Campbell, 2015; Warren et al., 2010), depression (Nuño et al., 2021), anxiety (Eysenck et al., 2007), and stress (Shields et al., 2016) will excessively deplete an individual’s executive functions and cognitive resources, making it difficult to allocate sufficient cognitive control to maintain CR. For instance, Fekete et al. (2021) found no significant indirect effects of self-compassion on restrained eating mediated by depression or anxiety. The authors attributed this null finding to the low prevalence of clinical-level depressive and anxiety symptoms among women in their sample. Consistent with this, Appleton and McGowan (2006) found no link between restrained eating and depression. Furthermore, research indicates that high-restraint women consume more high-fat foods than low-restraint women, regardless of stress levels (Habhab et al., 2009). Consequently, it is likely that the severe depletion of cognitive resources accounts for why none of the three negative emotions significantly mediated the association between attachment anxiety and CR.

Additionally, CR may be driven more by cognitive factors rather than emotional factors, such as the internalization of socio-cultural thin ideals (Martin-Wagar et al., 2025), perfectionism (Solomon-Krakus et al., 2025), and body dissatisfaction (Tang et al., 2021). Empirical evidence supports this inference. For example, Casale et al. (2025) found that parental overprotection (an early parenting style that may induce attachment anxiety) predicted restrained eating through the mediating roles of alexithymia and socially prescribed perfectionism. Additionally, Allan (2020) used a serial mediation analysis to show that attachment anxiety leads to more restrictive eating behaviors by lowering global self-esteem and in turn exacerbating the overvaluation of appearance. Accordingly, future research on CR should focus more on the role of cognitive factors (e.g., perfectionism, bias, negative evaluation).

Beyond the theoretical explanations, the nonsignificant indirect effects should also be interpreted considering the characteristics of the present data. Although the VIF values indicated no severe multicollinearity, depression, anxiety, and stress were highly correlated, and their shared variance may have reduced the unique contribution of each mediator in the parallel mediation model. In addition, the relatively low levels of depressive symptoms in the current sample may have limited variability in depression scores and weakened the detection of its potential mediating effect.

Despite these findings, several limitations of the present study should be acknowledged. First, the cross-sectional design limits the ability to establish causal relationships among attachment anxiety, negative emotions, and disordered eating behaviors. Although mediation analyses were conducted, the temporal sequence and causal pathways among these variables cannot be determined. Second, although age and BMI were included as covariates, other potentially confounding variables were not assessed in the present study. Previous studies have suggested that factors such as body dissatisfaction (Tang et al., 2021), previous health conditions (Pursey et al., 2020), self-esteem (Abdoli et al., 2025), perfectionism (Solomon-Krakus et al., 2025), emotion regulation difficulties (Dingemans et al., 2017), social comparison (Bonfanti et al., 2025), social media exposure (Dane & Bhatia, 2023), family environment (Scaglioni et al., 2018), and romantic relationship characteristics (Pepping et al., 2018; Gray & Dunlop, 2019) may also be associated with eating behaviors. In addition, BMI in the present study was calculated based on participants’ self-reported height and weight rather than objective anthropometric measurements, which may have introduced measurement errors. Third, the present study only included young female university students in China, which may limit the generalizability of the findings to males, non-university populations, clinical populations, or individuals from different cultural backgrounds.

Nevertheless, this study also has several strengths. First, by distinguishing among three types of disordered eating behaviors and simultaneously examining the differential mediating roles of depression, anxiety, and stress, this study provides a more detailed perspective for understanding the psychological mechanisms underlying the association between attachment anxiety and disordered eating. Second, this study employed a parallel multiple mediation model, which allowed the simultaneous estimation of three mediation pathways and controlled for the covariance among mediators, thereby enabling a more accurate evaluation of the unique mediating contributions of different negative emotions. Furthermore, age and BMI were included as covariates in the analytical model, and the use of well-validated measures, a relatively large sample size, and bootstrap confidence interval-based mediation analyses enhanced the reliability and robustness of the findings. Finally, this study has important practical implications, as the findings suggest that future interventions targeting attachment anxiety-related disordered eating problems may further focus on the management of anxiety and stress, providing theoretical support for the development of more precise psychological intervention strategies.

## 5. Conclusions

This study found that attachment anxiety significantly and positively predicted UE and EE but could not significantly predict CR. Moreover, anxiety and stress significantly mediated the relationships between attachment anxiety and both UE and EE, whereas depression showed no significant mediating effect. In addition, none of the three negative emotions showed a significant mediating effect between attachment anxiety and CR. These findings highlight the differential roles of negative emotions in the associations between attachment anxiety and distinct dimensions of disordered eating behaviors, providing further insights into the psychological mechanisms underlying disordered eating among young women.

Future research should adopt longitudinal designs to further clarify the temporal relationships and causal pathways among attachment anxiety, negative emotions, and disordered eating behaviors. In addition, future studies should incorporate more comprehensive psychological and sociocultural factors to further elucidate the mechanisms underlying the association between attachment anxiety and disordered eating. Furthermore, studies involving broader samples with diverse demographic characteristics and clinical backgrounds are needed to further validate the present findings and enhance their generalizability and external validity. Moreover, future research may employ latent variable structural equation modeling to explicitly account for measurement error and further validate the proposed mediation model at the latent construct level.

## Figures and Tables

**Table 1:** Participants’ demographic characteristics.

	Person (n)	Percent (%)
Total students	578	100%
**Age**		
18–19	332	57.44%
20–21	195	33.74%
22–23	39	6.75%
24–25	12	2.08%
**BMI**		
Underweight	123	21.28%
Normal weight	418	72.32%
Overweight	33	5.71%
Obese	4	0.69%
**Grade**		
First year	282	48.79%
Second year	73	12.63%
Third year	195	33.74%
Fourth year	28	4.84%
**Residence**		
Urban	154	26.64%
Rural	424	73.36%
**Ethnicity**		
Han	564	97.58%
Ethnic Minorities	14	2.42%
**Father’s education**		
Junior high school and below	84	14.53%
Senior high school	139	24.05%
College and above	355	61.42%
**Mother’s education**		
Junior high school and below	72	12.46%
Senior high school	103	17.82%
College and above	403	69.72%
**Monthly household income**		
<1000 RMB	22	3.81%
1000–3000 RMB	163	28.20%
3000–5000 RMB	201	34.78%
5000–8000 RMB	130	22.49%
>8000 RMB	62	10.73%

Note. n = number of participants. BMI = body mass index. According to the Chinese Public Health Standards, as follows: underweight, BMI < 18.5 kg/m^2^; normal weight, 18.5 ≤ BMI < 24.0 kg/m^2^; overweight, 24 ≤ BMI < 28.0 kg/m^2^; and obese, BMI ≥ 28.0 kg/m^2^.

**Table 2:** Descriptive statistics of key variables.

Variable	M	SD	SK	K
1. Age	19.48	1.48	1.14	1.40
2. BMI	20.25	2.27	0.90	1.64
3. Attachment anxiety	16.03	4.62	0.27	−0.23
4. Depression	2.10	3.21	2.78	9.96
5. Anxiety	2.88	3.35	2.11	6.00
6. Stress	3.42	3.59	1.61	3.47
7. Uncontrolled eating	41.34	21.00	0.34	−0.08
8. Emotional eating	39.66	27.59	0.32	−0.58
9. Cognitive restraint	39.46	17.73	0.10	−0.10

Note. M = mean; SD = standard deviation; SK = skewness; K = kurtosis; BMI = body mass index.

**Table 3:** Correlation analysis of key variables.

Variable	1	2	3	4	5	6	7	8
1. Age	1							
2. BMI	−0.05	1						
3. Attachment anxiety	0.01	0.03	1					
4. Depression	0.06	0.14 **	0.37 ***	1				
5. Anxiety	0.08 *	0.06	0.44 ***	0.76 ***	1			
6. Stress	0.09 *	0.12 **	0.43 ***	0.78 ***	0.83 ***	1		
7. Uncontrolled eating	0.03	0.14 **	0.28 ***	0.35 ***	0.39 ***	0.40 ***	1	
8. Emotional eating	0.07	0.19 ***	0.27 ***	0.35 ***	0.39 ***	0.40 ***	0.70 ***	1
9. Cognitive restraint	0.12 **	0.14 **	0.13 **	0.06	0.12 **	0.11 **	0.16 ***	0.19 ***

Note. BMI = body mass index; * *p* < 0.05, ** *p* < 0.01, *** *p* < 0.001.

**Table 4:** Mediating effect analysis of the parallel mediation model path.

Pathways	Est	SE	LLCI	ULCI
**Direct paths**				
Attachment anxiety → Depression	0.37	0.05	0.26	0.45
Attachment anxiety → Anxiety	0.44	0.05	0.33	0.52
Attachment anxiety → Stress	0.42	0.05	0.33	0.50
Depression → Uncontrolled eating	0.04	0.06	−0.09	0.16
Depression → Emotional eating	0.00	0.06	−0.11	0.13
Depression → Cognitive restraint	−0.12	0.08	−0.27	0.03
Anxiety → Uncontrolled eating	0.17	0.08	0.03	0.33
Anxiety → Emotional eating	0.18	0.07	0.04	0.33
Anxiety → Cognitive restraint	0.14	0.08	−0.02	0.30
Stress → Uncontrolled eating	0.17	0.08	0.02	0.32
Stress → Emotional eating	0.18	0.08	0.03	0.32
Stress → Cognitive restraint	0.02	0.08	−0.14	0.18
Attachment anxiety → Uncontrolled eating	0.12	0.05	0.03	0.21
Attachment anxiety → Emotional eating	0.11	0.04	0.03	0.20
Attachment anxiety → Cognitive restraint	0.10	0.05	−0.01	0.20
**Indirect paths**				
Attachment anxiety → Depression → Uncontrolled eating	0.01	0.02	−0.04	0.06
Attachment anxiety → Anxiety → Uncontrolled eating	0.07	0.03	0.01	0.15
Attachment anxiety → Stress → Uncontrolled eating	0.07	0.03	0.01	0.14
Attachment anxiety → Depression → Emotional eating	0.00	0.02	−0.04	0.05
Attachment anxiety → Anxiety → Emotional eating	0.08	0.03	0.02	0.15
Attachment anxiety → Stress → Emotional eating	0.07	0.03	0.01	0.15
Attachment anxiety → Depression → Cognitive restraint	−0.04	0.03	−0.11	0.01
Attachment anxiety → Anxiety → Cognitive restraint	0.06	0.04	−0.01	0.14
Attachment anxiety → Stress → Cognitive restraint	0.01	0.04	−0.06	0.08

Notes. Est = Standardized Estimate; SE = Standard Error; LLCI = Lower Limit of 95% Confidence Interval; ULCI = Upper Limit of 95% Confidence Interval.

## Data Availability

The datasets used during the current study are available from the corresponding author on reasonable request. The data are not publicly available due to privacy or ethical restrictions.

## Abbreviations

The following abbreviations are used in this manuscript:

UE	Uncontrolled Eating
EE	Emotional Eating
CR	Cognitive Restraint
RAAS	Revised Adult Attachment Scale
DASS-21	Depression, Anxiety and Stress Scale-21
TFEQ-18	Three-Factor Eating Questionnaire-18
BMI	Body Mass Index
VIF	Variance Inflation Factor
